# SUMOylation modulates FOXK2-mediated paclitaxel sensitivity in breast cancer cells

**DOI:** 10.1038/s41389-018-0038-6

**Published:** 2018-03-13

**Authors:** Gabriela Nestal de Moraes, Zongling Ji, Lavender Y.-N. Fan, Shang Yao, Stefania Zona, Andrew D. Sharrocks, Eric W.-F. Lam

**Affiliations:** 1 Department of Surgery and Cancer, Imperial College London, Imperial Centre for Translational and Experimental Medicine (ICTEM), Du Cane Road, London, W12 0NN UK; 2 Laboratório de Hemato-Oncologia Celular e Molecular, Programa de Hemato-Oncologia Molecular, Instituto Nacional de Câncer (INCA), Praça da Cruz Vermelha, 23/6° andar, Centro, 20230-130 Rio de Janeiro, Brazil; 3 Faculty of Biology, Medicine and Health, University of Manchester, Michael Smith Building, Oxford Road, Manchester, M13 9PT UK

## Abstract

The forkhead transcription factor FOXK2 plays a critical role in suppressing tumorigenesis and mediating cytotoxic drug action in breast cancer. However, the mechanism by which the biological function of FOXK2 is regulated remains poorly understood. Here, we investigated the role of SUMOylation in modulating FOXK2-mediated drug sensitivity. We identified SUMOylation consensus motifs within the FOXK2 sequence and constructed two SUMOylation-defective double mutants by converting lysine 527 and 633 to arginines and glutamic acid 529 and 635 to alanines, respectively. We found that both the FOXK2 SUMOylation-deficient (K527/633 R) and (E529/635 A) mutants were ineffective in mediating the cytotoxic function of paclitaxel when compared to the wild-type (WT) FOXK2. When overexpressed, unlike the wild-type (WT) FOXK2, the K527/633 R mutant had little effect on the sensitivity of MCF-7 and MDA-MB-231 cells to paclitaxel, as examined by cell viability and clonogenic assays. Our results also showed that MCF-7 cells overexpressing the K527/633 R mutant form of FOXK2 or the empty expression vector have lower protein and mRNA levels of its tumour suppressive transcriptional target FOXO3 compared to the wild-type FOXK2. Consistently, ChIP assays revealed that unlike wild-type FOXK2, the SUMOylation-defective (K527/633 R) mutant is unable to bind to the *FOXO3* promoter, despite expressing comparable levels of protein and having the same subcellular localization as the wild-type FOXK2 in MCF-7 cells. Interestingly, expression of neither the wild-type nor the K527/633 R mutant FOXK2 had any effect on the proliferation and paclitaxel sensitivity of the MCF-7 Tax^R^ paclitaxel-resistant cells. In agreement, both the wild-type and the (K527/633 R) mutant FOXK2 failed to bind to the endogenous *FOXO3* promoter in these cells. Collectively, our results suggest that SUMOylation positively regulates FOXK2 transcriptional activity and has a role in mediating the cytotoxic response to paclitaxel through the tumour suppressor FOXO3.

## Introduction

Forkhead box K2 (FOXK2) belongs to the family of forkhead transcription factors, which share a conserved DNA binding domain^1^ and regulate a wide spectrum of biological processes within the cell^2,3^. Despite being first uncovered in 1991 as an NFAT-like interleukin^4^, the biological function of FOXK2 remains enigmatic. FOXK2 has been shown to regulate gene networks, including those involved in cancer^5^ and to interact with subunits of the polycomb complex, recruiting the BRCA1-associated protein to target genes and, thus, involve in chromatin remodeling^6^. In breast cancer, FOXK2 has been demonstrated to downregulate ERα protein stability and transcriptional activity, resulting in decreased cell growth^7^. Subsequently, a negative role of FOXK2 in breast cancer development and progression has been established, in which FOXK2 transcriptionally represses genes involved in cell cycle, DNA damage response, p53 and hypoxia pathways by directly interacting with multiple transcription co-repressor complexes^8^. Accordingly, depletion of FOXK2 is associated with tumorigenesis and aggressive features in breast cancer in vitro and in vivo, pointing to FOXK2 as a potential tumour suppressor^8^. Recently, FOXK2 has also been implicated in mediating breast cancer drug action. Accordingly, FOXK2 mediates drug sensitivity in breast cancer cells in a FOXO3-dependent fashion^9^. On the other hand, FOXK2 accumulates in the nucleus of drug resistant cells, but fails to transcriptionally activate FOXO3 expression, suggesting that it might be regulated at the post-translational level.

SUMOylation is a post-translational modification essential for multiple cellular processes such as DNA damage response, nuclear-cytoplasmic shuttle, cell cycle, apoptosis, protein stability and transcriptional regulation^10^. SUMOylation is a reversible process involving conjugation and de-conjugation of SUMO proteins, which target lysines in a consensus sequence (*ψ*KxD/E, where *ψ* is a hydrophobic amino acid)^11^. Given that FOX transcription factors have been shown to have their expression and transcriptional activity modified by SUMOylation^12–14^, we hypothesized that SUMOylation could modulate FOXK2 function in drug resistance. Here, we report that FOXK2 is modified by SUMOylation and overexpression of a SUMOylation-defective form of FOXK2 prevents endogenous FOXK2-mediated induction of FOXO3 expression and confers paclitaxel resistance to drug-sensitive breast cancer cells. Conversely, SUMOylation of FOXK2 appears to be impaired in paclitaxel-resistant cells, suggesting that SUMOylation might act to enhance FOXK2 transcriptional activity and, thus, FOXK2-mediated paclitaxel sensitivity in breast cancer^9^.

## Results

### FOXK2 is modified by SUMOylation

**Fig. 1:**
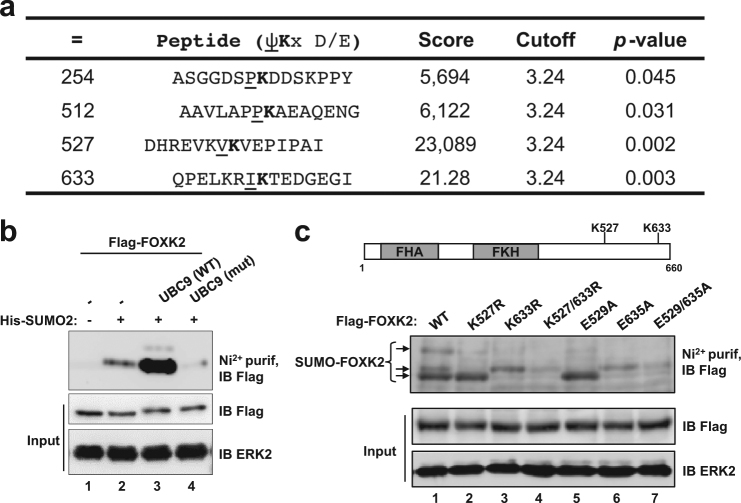
FOXK2 is modified by SUMOylation. **a** FOXK2 consensus sequences for SUMOylation motifs identified through the GPS-SUMO web server (http://sumosp.biocuckoo.org/online.php). **b** HEK293T cells were transfected with plasmids encoding Flag-tagged FOXK2 and, where indicated, His-tagged SUMO2, wild-type (WT) UBC9 or the catalytically inactive mutant (mut) UBC9. SUMO-modified FOXK2 was identified by purification using Ni + affinity beads followed by detection by immunoblotting (IB) with anti-Flag antibody (top). Input levels of FOXK2 and ERK2 loading control were detected by IB (bottom). **c** Top: A schematic representation of the domain structure and location of the putative consensus SUMOylation sites in human FOXK2. Bottom: HEK293T cells were transfected wild-type (WT) FOXK2 or the indicated SUMO site mutants along with His-tagged SUMO2 and constitutively active MEK to enhance SUMOylation levels. SUMO-modified FOXK2 was identified by purification using Ni + affinity beads followed by detection by immunoblotting (IB) with anti-Flag antibody (top). Input levels of FOXK2 and ERK2 loading control were detected by IB (bottom)

FOXK2 expression has been shown to be induced upon drug treatment and to mediate paclitaxel sensitivity;^9^ however, manipulation of FOXK2 levels through knockdown or overexpression experiments could not modulate the drug resistance phenotype of MCF-7 Tax^R^ paclitaxel-resistant cells^9^. Considering that FOXK2 accumulates in the nucleus of drug-resistant cells, we speculated that FOXK2 activity might be regulated by post-translational modifications (PTMs). To gain some insights into the role of PTMs in paclitaxel response, we initially focused on the role of SUMOylation in modulating FOXK2 activity. To this end, we first screened for putative SUMOylation sites in the FOXK2 sequence, using a web-based prediction tool (http://sumosp.biocuckoo.org/online.php)^15^. Based on analysis with GPS-SUMO, two consensus SUMOylation motifs (aa positions: 527 and 633) were identified within the FOXK2 sequence that show high scores and statistically significant *p* values (Fig. 1a). To establish whether FOXK2 could be modified by SUMO at these two residues, we co-transfected FOXK2 with His-tagged SUMO2 and isolated SUMO-conjugated proteins by nickel affinity purification under denaturing conditions followed by detection of FOXK2 by Western blotting. FOXK2 was only pulled down and detected by Western blotting in the presence of co-transfected His-tagged SUMO (Fig. 1b, lane 2). Furthermore, co-transfection with the wild-type UBC9 SUMO conjugating enzyme caused an increase in the levels of SUMO-modified FOXK2, but no such increase was observed with a catalytically inactive version of UBC9 (Fig. 1b, lanes 3 and 4). Next, we asked whether either K527 and/or K633 in FOXK2 are the sites for SUMO conjugation by mutating them either individually or in combination. We also created mutations at the acidic residues in the ΦKXE motif. Individual lysine mutations resulted in decreased levels of SUMO conjugates and combinatorial mutation virtually abolished SUMO conjugation (Fig. 1c, lanes 2–4). Similar results were obtained with glutamic acid mutations, where combinatorial mutation again resulted in greatly reduced SUMO conjugation (Fig. 1c, lanes 5–7). Collectively, these data demonstrate that FOXK2 can be modified by SUMOylation and that K527 and K633 are the major sites for SUMO conjugation.

### Overexpression of a SUMO-mutant form of FOXK2 partially impairs FOXK2-mediated paclitaxel sensitivity in breast cancer cells

**Fig. 2:**
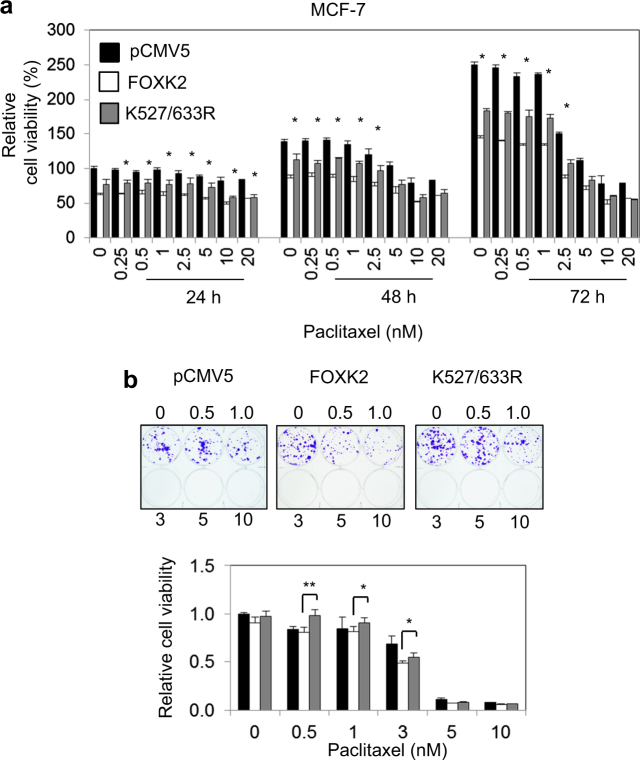
FOXK2 activity is stimulated by SUMOylation. **a** MCF-7 cells were transfected with the empty vector (pCMV5), wild-type FOXK2 and SUMO-mutant FOXK2 vector (K527/633 R), seeded in 96-well plates and treated with increasing concentrations of paclitaxel. After 24, 48 and 72 h of incubation with the drugs, cell viability was assessed by SRB assay. Bars represent average ± s.d. from three independent experiments. Statistical significant differences between cells transfected with the wild-type and K527/633 R were determined by Student’s *t*-test (*p ≤ 0.05; **p ≤ 0.01; ***p ≤ 0.001, significant). **b** MCF-7 cells were transfected with the empty vector (pCMV5), wild-type FOXK2 and SUMO-mutant FOXK2 vector (K527/633 R), seeded in 6-well plates and treated with increasing concentrations of paclitaxel. After 48 h of incubation with the drugs, cells were cultured in fresh media, grown for around 14 days and stained with crystal violet. The graphs are representative of three experiments. Statistical significant differences between cells transfected with the wild-type and K527/633 R were determined by Student’s *t*-test (*p ≤ 0.05; **p ≤ 0.01; ***p ≤ 0.001, significant)

**Fig. 3:**
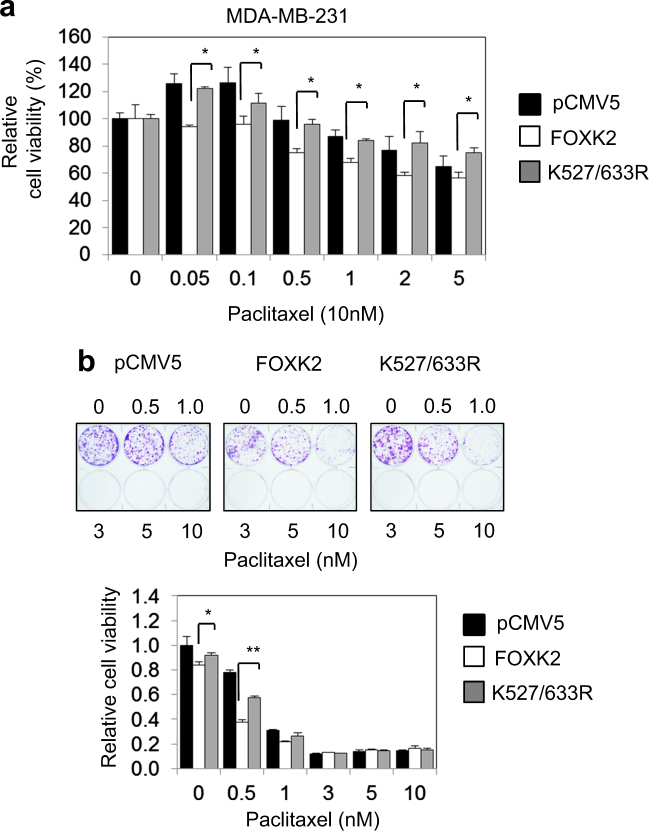
SUMOylation affects FOXK2 activity also in MDA-MB-231 cells. MDA-MB-231 cells were transfected with the empty vector (pCMV5), wild-type FOXK2 and SUMO-mutant FOXK2 vector (K527/633 R) and treated with increasing concentrations of paclitaxel, after which cell viability was assessed by SRB assay (**a**) and proliferation by clonogenic assay (**b**). The graphs are representative of three experiments. Statistical significant differences between cells transfected with the wild-type and K527/633 R were determined by Student’s *t*-test (*p ≤ 0.05; **p ≤ 0.01; ***p ≤ 0.001, significant)

Next, we tested the role of FOXK2 SUMOylation in mediating the cytotoxic function of paclitaxel in breast cancer cells. To this end, MCF-7 cells were transfected with either the empty expression vector, the wild-type FOXK2 (WT), or the FOXK2 (K527/633 R) mutant and their sensitivity to paclitaxel analysed through short and long-term cell viability assays. The results showed that MCF-7 cells transfected with the SUMO-defective FOXK2 mutant K527/633 R is less sensitive to paclitaxel than MCF-7 cells transfected with wild-type FOXK2 (Fig. 2a). Consistently, MCF-7 cells transfected with the K527/633 R mutant FOXK2 had the capacity to form more colonies in untreated and paclitaxel-treated cells compared to the cells transfected with wild-type FOXK2 (Fig. 2b), suggesting that SUMOylation of FOXK2 at these sites mediates the cytotoxic effects of paclitaxel. In addition, we also repeated the clonogenic assays with the combinational glutamic acid mutation (E529/635 A). In concordance, like the K527/633 R mutant, the FOXK2 SUMOylation-deficient E529/635 A mutant was also ineffective in mediating the cytotoxic function of paclitaxel when compared to the wild-type (WT) FOXK2 in clonogenic assays, further confirming that FOXK2 SUMOylation at K527/633 mediates the cytotoxic effects of paclitaxel (Supplementary Fig. S1). We next performed this set of experiments with the K527/633 R mutant FOXK2 in the triple negative MDA-MB-231 breast carcinoma cells and again found the K527/633 R mutant form of FOXK2 is defective in conferring the cytotoxic/cytostatic functions of paclitaxel in MDA-MB-231 cells (Figs. 3a, b). Collectively, these results suggest that FOXK2 SUMOylation is required for the cytotoxic function of paclitaxel.

### The expression of FOXO3 is downregulated in K527/633R FOXK2-overexpressing cells

**Fig. 4:**
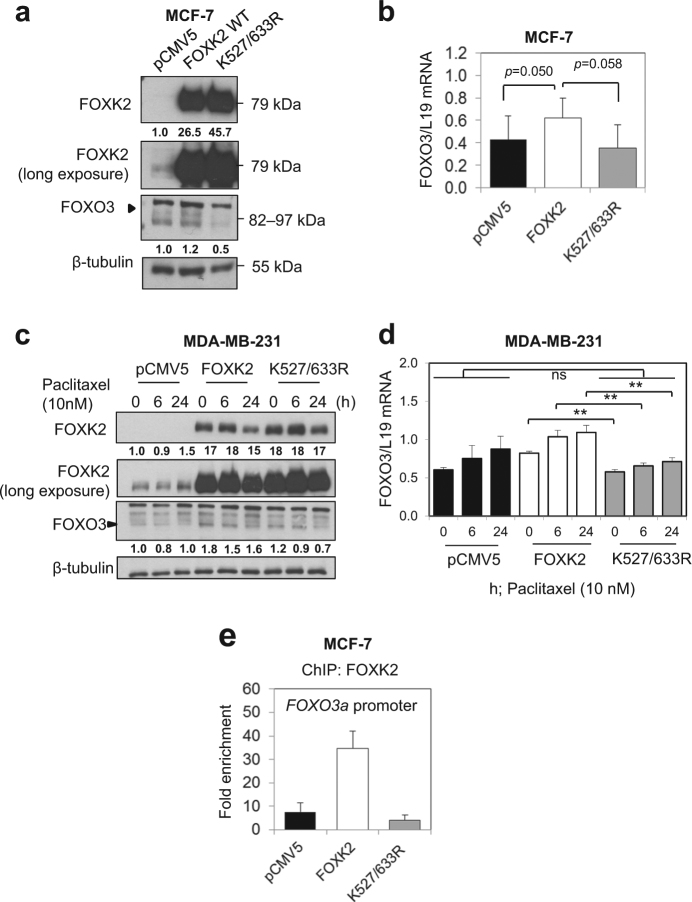
Overexpression of a SUMO-mutant form of FOXK2 partially impairs FOXK2-mediated FOXO3 regulation. MCF-7 cells were transfected with the empty vector (pCMV5), wild-type FOXK2 and SUMO-mutant FOXK2 vector (K527/633 R) and collected for analysis of FOXO3 expression by western blot (**a**) and qRT-PCR (**b**). Following transfection with the empty vector (pCMV5) wild-type FOXK2 and SUMO-mutant FOXK2 vector (K527/633 R), MDA-MB-231 cells were treated with 10 nM paclitaxel for the indicated times and subjected to western blot analysis, where FOXO3 levels were determined by Western blot (**c**) and qRT-PCR (**d**). For 4a and 4c, the relative expression levels of FOXK2 and FOXO3 were determined based on the expression levels of the target gene product versus the reference, Tubulin, and the values shown under the respective western blot bands. The intensities of the unsaturated western blot bands were determined using the ImageJ software. Bars represent average ± s.d. of three independent experiments. Statistical significance was determined by Student’s *t*-test (*p ≤ 0.05; **p ≤ 0.01; ***p ≤ 0.001, significant; ns = not significant). **e** MCF-7 transfected with the empty vector (pCMV5), wild-type FOXK2 and SUMO-mutant FOXK2 vector (K527/633 R) were harvested for chromatin immunoprecipitation assays using the IgG as negative control and anti-FOXK2 antibody. After reversal of cross-linking, the coimmunoprecipitated DNA was amplified by qRT-PCR, using primers amplifying the FOXK2 binding-site containing region in the *FOXO3* promoter

The FOXO3 transcription factor has been shown to mediate the cytotoxic effects of chemotherapeutic agents in breast cancer through promoting the expression of downstream transcriptional targets involved in proliferative arrest and cell death^9^. In addition, we have also demonstrated that FOXO3 plays a key role in mediating the cytotoxic function of FOXK2 in response to paclitaxel in breast cancer cells^9^. To further confirm our findings on the regulation of *FOXO3* gene expression by FOXK2 and to investigate how this can be affected by FOXK2 SUMOylation, we evaluated FOXO3 expression and carried out ChIP of the *FOXO3* promoter region in MCF-7 cells transfected with the wild-type and K527/633 R mutant FOXK2. Interestingly, FOXO3 protein and mRNA levels were upregulated in MCF-7 cells transfected with the wild-type FOXK2 but not with K527/633 R mutant or the empty vector (Figs. 4a, b). Accordingly, the protein and mRNA expression levels of FOXO3 were also found to be lower in the MDA-MB-231 cells transfected with the empty vector and K527/633 R mutant compared to the wild-type FOXK2 (Figs. 4c, d; Supplementary Figure S2). The overexpression of wild-type and mutant FOXK2 was validated by qRT-PCR in MDA-MB-231 cells (Supplementary Figure S2a). Following paclitaxel treatment, FOXO3 mRNA levels remained fairly stable in the pCMV5 and K527/633 R mutant transfected MDA-MB-231 cells, in contrast with what we observed with cells transfected with wild-type FOXK2 vectors (Fig. 4d; Supplementary Figure S2b). In agreement, overexpression of wild-type FOXK2 resulted in a dramatic increase in FOXK2 binding to the *FOXO3* promoter, while no such increases were observed in the MCF-7 cells transfected with the K527/633 R mutant (Fig. 4e). Collectively, these results suggest that SUMOylation enhances FOXK2 recruitment to its target genes, including *FOXO3*, and its ability to promote its target gene activation.

### Recruitment of FOXK2 to the endogenous *FOXO3* promoter is impaired in paclitaxel-resistant breast cancer cells

**Fig. 5:**
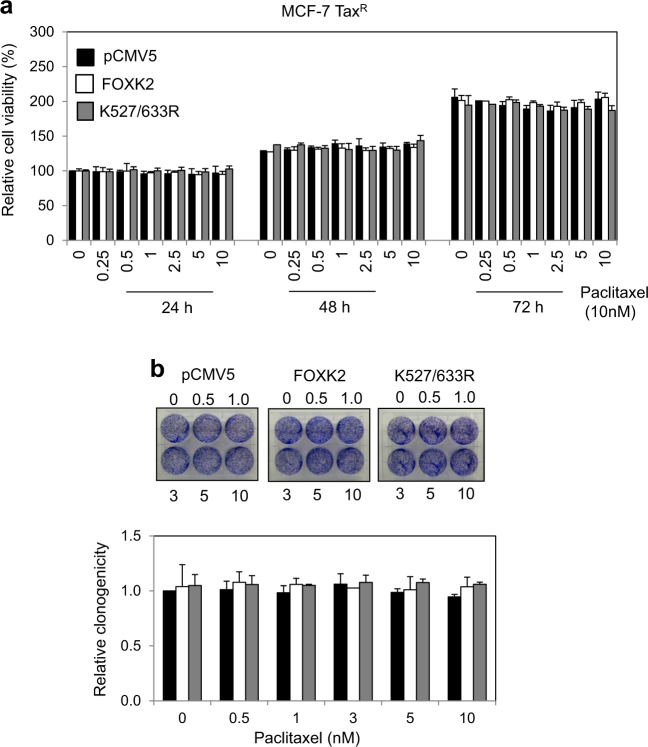
FOXK2 SUMOylation is impaired in paclitaxel-resistant cells. **a** MCF-7 Tax^R^ cells were transfected with the empty vector (pCMV5), wild-type FOXK2 and SUMO-mutant FOXK2 vector (K527/633 R), seeded in 96-well plates and treated with increasing concentrations of paclitaxel. After 24, 48 and 72 h of incubation with the drugs, cell viability was assessed by SRB assay. Bars represent average ± s.d. from three independent experiments. Statistical significance was determined by Student’s *t*-test (*p ≤ 0.05; **p ≤ 0.01; ***p ≤ 0.001, significant). **b** MCF-7 Tax^R^ cells were transfected with the empty vector (pCMV5), wild-type FOXK2 and SUMO-mutant FOXK2 vector (K527/633 R), seeded in 6-well plates and treated with increasing concentrations of paclitaxel. After 48 h of incubation with the drugs, cells were culture in fresh media, grown for around 14 days and stained with crystal violet. The graphs are representative of three experiments. Statistical significance was determined by Student’s *t*-test (*p ≤ 0.05; **p ≤ 0.01; ***p ≤ 0.001, significant)

**Fig. 6:**
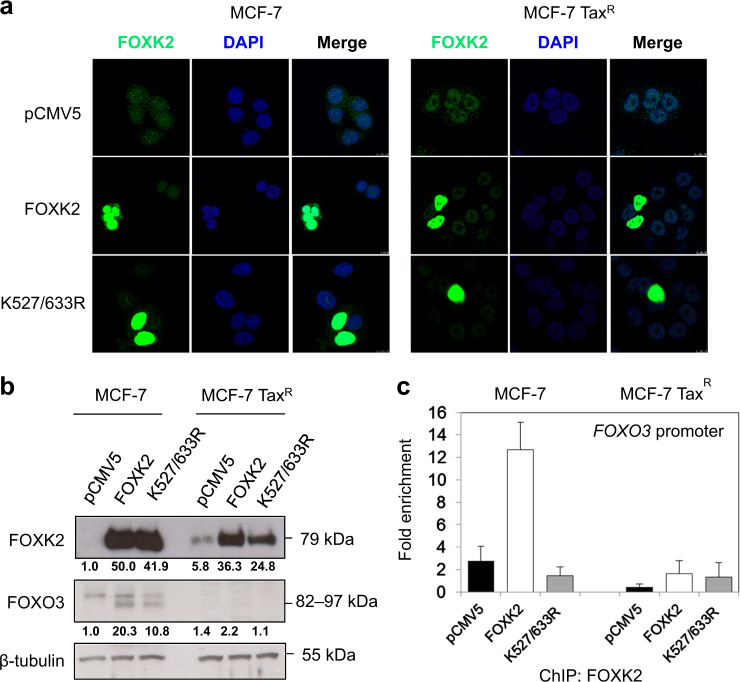
SUMOylation of FOXK2 affects binding to FOXO3 promoter gene. **a** FOXK2 is localized at the nucleus of paclitaxel-sensitive and resistant cells overexpressing the K527/633 R mutant form of FOXK2. MCF-7 (left panel) and MCF-7 TaxR (left panel) cells were transfected with the empty vector (pCMV5), wild-type FOXK2 vector, and SUMO-mutant vector (K527/633 R) for 24 h and seeded on chamber slides. Cells were then fixed and immunostained for FOXK2 (green). Nuclei were counterstained with 4′-6-diamidino-2-phenyllindole (DAPI; blue). Images were acquired with Leica TCS SPS (x63 magnification). Images are representative of two independent experiments. **b** MCF-7 and MCF-7 TaxR cells were transfected with the empty vector (pCMV5) wild-type FOXK2 and SUMO-mutant FOXK2 vector (K527/633 R) and subjected to western blot analysis, where FOXK2, FOXO3 and β-tubulin levels were determined. The the relative expression levels of FOXK2 and FOXO3 were determined based on the expression levels of the target gene product versus the reference, Tubulin, and the values shown under the respective western blot bands. The intensities of the unsaturated western blot bands were determined using the ImageJ software. **c** MCF-7 and MCF-7 TaxR cells transfected with the empty vector (pCMV5), wild-type FOXK2 and SUMO-mutant FOXK2 vector (K527/633 R) were used for chromatin immunoprecipitation assays using the IgG as negative control and anti-FOXK2 antibody. After reversal of cross-linking, the coimmunoprecipitated DNA was amplified by qRT-PCR, using primers amplifying the FOXK2 binding-site containing region in the *FOXO3* promoter

Next, we investigated whether FOXK2 SUMOylation has a role in modulating drug sensitivity in the paclitaxel-resistant MCF-7 Tax^R^ cells. To address this, we overexpressed the wild-type and K527/633 R mutant FOXK2 and assessed the cell viability by proliferative (Fig. 5a) and clonogenic assays (Fig. 5b). We found that overexpression of neither the K527/633 R mutant nor the wild-type FOXK2 had any effects on the proliferation and clonogenicity of the MCF-7 Tax^R^ cells (Fig. 5a, b). It is known that SUMOylation can affect the subcellular localization of its targets^16^, so we analysed FOXK2 expression and localization of the K527/633 R mutant and the wild-type FOXK2 by confocal microscopy. We observed that FOXK2 was localized mainly at the nucleus of cells transfected with the control pCMV5, the wild-type and K527/633 R FOXK2 in both MCF-7 and MCF-7 Tax^R^ cells (Fig. 6a). This result indicates that the effects observed following transfections with mutant FOXK2 are not due to FOXK2 mislocalisation to the cytoplasm. To gain further insights into the FOXK2-mediated regulation of its downstream target FOXO3, we performed immunoblotting in control, the wild-type FOXK2 and K527/633 R mutant-transfected MCF-7 Tax^R^ cells and found that, in contrast with the parental MCF-7 cells, FOXO3 expression levels were not induced by overexpression of the wild-type and K527/633 R mutant FOXK2 (Fig. 6b). In concordance, the FHRE region of endogenous *FOXO3* promoter was not occupied by the transfected wild-type and K527/633 R mutant FOXK2 in MCF-7 Tax^R^. This is in contrast to the MCF-7 cells, in which strong binding to *FOXO3* promoter is clearly observed with the transfected wild-type FOXK2, but the recruitment was disrupted with the transfected mutant FOXK2 (Fig. 6c). Altogether, our findings suggest that FOXK2 and its SUMOylation is crucial for the its recruitment to the *FOXO3* promoter, which in turn controls the cytotoxic function of paclitaxel in breast cancer cells and that the recruitment of FOXK2 to the *FOXO3* promoter is impaired in paclitaxel-resistant breast cancer cells.

## Discussion

Breast cancer is a leading cause of deaths in women worldwide. This is partly explained by the acquisition of chemotherapeutic drug resistance, which accounts for poor responses and eventually, relapse and therapeutic failure. The FOXK2 transcription factor has been implicated in cancer drug resistance, where it has been shown to modulate sensitivity to paclitaxel and epirubicin via inducing the expression of FOXO3^9^. We have also shown that FOXK2 function is deregulated in drug-resistant breast cancer cells. However, little is known about the mechanism by which FOXK2 expression and function is regulated. In this study, we investigated the role of SUMOylation in the regulation of FOXK2 and its role in modulating drug sensitivity in breast cancer. We identified a number of putative SUMOylation consensus residues in FOXK2 and used this information to generate a SUMOylation defective mutants. We found that, compared with the wild-type FOXK2, both the SUMO-mutants (i.e., K527/633 R and E529/635 A) are defective in mediating the paclitaxel-induced cytotoxicity in breast cancer cells. We also observed that ectopic overexpression of the SUMO-defective FOXK2 mutant can at times reduce paclitaxel-induced cytotoxicity in cell viability and clonogenic assays, and this is probably mediated through titrating away the activity of endogenous wild-type FOXK2. We do not exclude the fact that other upstream post-translational modifications may facilitate FOXK2 SUMOylation. In this manner, overexpression of the SUMO-deficient FOXK2 mutant will soak up these post-translational modification signals from the endogenous FOXK2 and cause an apparent downregulation of FOXO3 expression, especially upon paclitaxel treatment. In addition, breast cancer cells overexpressing the mutant form of FOXK2 had substantial lower protein and mRNA expression levels of FOXO3 when compared with wild-type FOXK2. Appropriately, FOXK2 has previously been reported to mediate the cytotoxic function of paclitaxel through regulating the expression of the FOXO3 transcription factor in breast cancer cells^9^, while FOXO3, in turn, is essential for mediating the effects of paclitaxel in breast cancer cells through driving the expression of downstream apoptotic target genes, such as Bim^17,18^. The moderate induction of FOXO3 by overexpression of wild-type FOXK2 is likely due to the fact that although ectopic overexpression of FOXK2 can result in a net increase in the levels of SUMOylated FOXK2, the steady state free SUMO and/or SUMOylation signals available to mediate FOXK2 SUMOylation is limiting. Treatment of paclitaxel makes more free SUMO available and/or increases SUMOylation signals to promote further FOXK2 SUMOylation. FOXO3 can also mediate the cytotoxic and cytostatic effects of paclitaxel indirectly through repressing the expression and activity of another Forkhead transcription factor FOXM1^19^. Accordingly, paclitaxel has been shown to target the expression of FOXM1 and its downstream target the kinesin KIF20A, to drive abnormal mitotic spindle formation, mitotic catastrophe and senescence in breast cancer cells^20^. Notably, these cytotoxic effects of FOXK2 were observed in both MCF-7 and MDA-MB-231 cell lines, which resemble different breast cancer molecular subtypes, suggesting that this mechanism of FOXK2 regulation is independent of expression of estrogen, progesterone and Her2 receptors. Evidence from ChIP experiments shows that the recruitment of wild-type and mutant FOXK2 to the *FOXO3* promoter is disrupted in the paclitaxel resistant MCF-7 Tax^R^ cells. In agreement, the expression levels of FOXO3 are substantially lower in the paclitaxel-resistant cells^9,17,18^. We have also obtained evidence previously which suggests that FOXO3 expression is downregulated in the MCF-7 TaxR cells at both the transcriptional and the post-translational levels^9,17,18^. This might be the reason behind why FOXK2 SUMOylation, which only affects FOXO3 transactivation, has little effect on FOXO3 expression in the established resistant cells. Interestingly, both the wild-type and the mutant (K527/633 R) FOXK2 accumulated in the nucleus of the MCF-7 Tax^R^ transfected cells, indicating that the lack of FOXK2 recruitment to the target genes is not due to an alteration in the subcellular distribution, a biological process commonly controlled by SUMOylation. SUMOylation is frequently enhanced upon cell stress, indicating that SUMOylation could represent an anti-proliferative cellular response^21^. Since drug treatment triggers intracellular signalling cascades associated with genotoxic stress and DNA damage response, it is plausible that mutation to SUMOylation sites could disrupt cellular response to drug treatment. Taken together, our findings suggest that inhibition of SUMOylation modulates the role of FOXK2 in paclitaxel response and suppresses its transcriptional activity of FOXK2 in breast cancer cells.

Our results also show that overexpression of neither the wild-type nor the SUMO-mutant FOXK2 has any effects on cell viability or colony formation in the paclitaxel resistant MCF-7 Tax^R^ cells. In agreement, ChIP analysis demonstrated that both the wild-type and mutant FOXK2 could not bind to the endogenous *FOXO3* promoter. However, this was not due to the mislocalization of FOXK2, as both the transfected wild-type and mutant FOXK2 accumulated in the nucleus. These data further confirm that FOXK2 cannot be recruited to target genes in paclitaxel-resistant cells. It is likely that epigenetic changes could interfere with the recruitment of FOXK2 to the chromatin or that FOXK2 function could be under the control of other post-translational modifications, which is deregulated in paclitaxel-resistant cells. FOXK2 has been demonstrated to be phosphorylated by cyclin/CDK complexes in a cell-cycle dependent manner^22^. Moreover, CDK-induced hyperphophorylated FOXK2 is less stable and loses transcriptional activity, suggesting that it might inactivate FOXK2 function. Nevertheless, the role of FOXK2 phosphorylation by CDK/cyclin complexes and other kinases on the regulation of drug resistance in cancer remains to be elucidated.

SUMOylation has been shown to regulate a wide range of crucial biological processes, which also include transcription^10,23^. SUMOylation of transcription factors and co-factors is involved in their regulation of gene expression; however, it can have opposing effects on their transcriptional activity. Although transcriptional regulation attributed to SUMOylation has been shown generally to be inhibitory in most cases, SUMOylation of some transcription factors can also promote transcription activation^23^. Here, we found that overexpression of a SUMO-defective plasmid impairs the endogenous FOXK2 function in transcription and drug sensitivity in breast cancer cells, confirming FOXK2 as a transcription factor whose transcriptional activity can be stimulated by SUMOylation. This finding contrasts reports with FOXM1^12^, FOXC2^13^ and FOXA1^14^, whose transcriptional activity is negatively regulated by SUMOylation. It indicates that SUMOylation of different members of the superfamily of FOX transcription factors may result in distinct outcomes, strengthening our current knowledge on how these factors are regulated by post-translational modifications. A question that still remains to be addressed is the role of the single mutants K527R and K633R on FOXK2-induced drug sensitivity. Additionally, it would be of importance to examine whether these FOXK2 residues are found SUMOylated in breast cancer patients. Regardless, we identified lysines 527 and 633 in combination as key FOXK2 sites potentially regulated by SUMOylation in vitro.

In conclusion, our study demonstrates that FOXK2 SUMOylation plays a crucial role in mediating the cellular cytotoxic function of paclitaxel in breast cancer cells. In addition, our work reveals that FOXK2 SUMOylation modulates its transcriptional activity in response to paclitaxel and that paclitaxel resistance is associated with the inability of FOXK2 to bind target genes, including the tumour suppressor FOXO3.

## Materials and methods

### Cell culture

MCF-7 and MDA-MB-231 human breast cancer cell lines were obtained from the American Type Culture Collection (Manassas, VA, USA) and were acquired from the Cell Culture Service from Cancer Research UK, where they were authenticated. The paclitaxel-resistant MCF-7 Tax^R^ cell line originated from MCF-7 and maintained in 50 nM paclitaxel^24^. All cell lines, including HEK293T, were cultured in Dulbecco’s modified Eagle’s medium supplemented with 10% foetal calf serum, 100 U/ml penicillin/streptomycin and 2 mM glutamine and maintained in a humidified incubator with 10% CO_2_ at 37 °C.

### Plasmid construction

His6-SUMO-2 expression vectors and Ubc9 and its C93S mutant expression plasmids were kindly provided by Ron Hay. pAS2252 (pCMV-driven construct encoding full-length FLAG-tagged human FOXK2) has been described previously^22^. The following plasmids encoding FOXK2 with mutations in lysine residues or glutamate residues, were made by Quikchange mutagenesis using the primer-template combinations: pAS2527 encoding FOXK2(K527R) (ADS2027/ADS2028-pAS2252), pAS2528 encoding FOXK2(K633R) (ADS2029/ADS2030-pAS2252), pAS2532 encoding FOXK2(K527/K633R) (ADS2029/ADS2030-pAS2527), pAS2529 encoding FOXK2(E529A) (ADS2031/ADS2032-pAS2252), pAS2530 encoding FOXK2(E635A) (ADS2033/ADS2034-pAS2252) and pAS2531 encoding FOXK2(E529/E635A) (ADS2033/ADS2034-pAS2529), respectively. The FuGENE 6 reagent (Roche, IN, USA) was used for plasmid transfections, according to manufacturers’ instructions. Following 24 h of transfection, cells were trypsinised and seeded for drug treatment.

### Sulforhodamine B (SRB) assay

The sulforhodamine B assay was performed for analysis of short-term alterations in cell viability in drug-treated cells following FOXK2 overexpression. Briefly, 3000 cells were seeded in 96-well plates and left to adhere for 24 h, after which paclitaxel was added. Cells were maintained in culture for additional 24, 28 and 72 h, fixed with 100 µl of 40% trichloroacetic acid for 1 h at 4 °C, washed 3 times with distilled water and stained with 100 µl SRB solution (0.4% SRB diluted in 1% acetic acid) for 1 h. Afterwards, plates were washed 3 times with 1% acetic acid and air-dried. Protein-bound dye was solubilized in 100 µl of 10 mM Tris solution and optical density was measured in a microplate reader at 492 nm (Sunrise, Tecan, Dorset, UK).

### Clonogenic assay

Following FOXK2 overexpression, a total of 2000 MCF-7, MCF-7 Tax^R^, or MDA-MB-231 cells were seeded into six-well plates and left overnight for adherence, after which they were treated with increasing concentrations of paclitaxel. After 48 h of incubation with the drug, cells were cultured in fresh drug-free media and grown for around 14 days until colony formation. Colonies were washed 3 times with PBS and fixed with 4% formaldehyde for 15 min at room temperature. After 3 additional washes with PBS, colonies were stained with 0.5% crystal violet (Sigma Aldrich; St. Louis, Missouri, USA) for 1 h, washed with flowing water and air-dried. Then, 1 ml of 33% acetic acid was added to each well for crystal violet solubilization. Optical density was measured at 592 nm using a microplate reader (Sunrise, Tecan, Dorset, UK).

### Confocal microscopy

For immunostaining, cells overexpressing pCMV5-FOXK2WT, pCMV5-FOXK2 K527/633 R or the empty vector pCMV5 were cultured in chamber slides. Afterwards, cells were fixed with 4% paraformaldehyde (Thermo Scientific, Rockford, IL, USA) for 15 min and permeabilised for 10 min with 0.2% Triton X-100, after which blocking with 5% goat serum for 30 min at room temperature was performed. The slides were then incubated overnight at 4 °C with FOXK2 antibody (A301-729A; Bethyl), washed with PBS and incubated with a 1:500 dilution of Alexa-Fluor 555-conjugated goat anti-rabbit secondary antibody (Molecular Probes, Invitrogen) for 45 min at room temperature. Subsequently, cells were washed with PBS and mounted with Vectashield mounting solution containing DAPI (4′-6-diamidino-2-phenyllindole; Vector Laboratories), prior to visualization with a Leica TCS SP5 confocal microscope (Leica Microsystems, Mannhein, Germany) equipped with a 63 × oil immersion objective and LAS-AF software.

### Western blotting and nickel affinity precipitation

For Western blotting, whole cell extracts were prepared by lysing cells in lysis buffer as previously described^25^ and 15 µg of protein were loaded. The antibodies recognizing FOXK2 (A301-729A), total FOXO3 (07-702) and β-tubulin (sc-9104) were purchased from Bethyl, Millipore and Santa Cruz Biotechnology, respectively. Primary antibodies were detected using horseradish peroxidase-linked anti-rabbit conjugate (Dako Glostrup, Denmark) and visualized using the ECL detection system (PerkinElmer, Waltham, Massachussets, USA).

His-tagged SUMO2 conjugated proteins were precipitated using nickel affinity beads as described previously^26^. Western blotting was performed with anti-Flag M2 (F3165, Sigma), and anti-ERK2 (sc-154, Santa Cruz Biotechnology) primary antibodies in combination with Infrared IRDye-labelled secondary antibodies and bands detected using a Li-Cor Odyssey infrared imager.

### Quantitative real time PCR (qRT-PCR)

Total RNA extraction was performed with the RNeasy Mini Kit (Qiagen, Hilden, Germnay). Complementary DNA was synthetized by the Superscript III Method (Invitrogen, Paisley, UK), according to manufacturers’s instructions. FOXO3 transcript levels were analysed by qRT-PCR using the standard curve and normalized to ribosomal protein L19 mRNA levels, as previously described^27^. The forward and reverse primers used were: L19-F: 5′-GCGGAAGGGTACAGCCAAT-3′ and L19-R: 5′-GCAGCCGGCGCAAA-3′, and FOXO3-F: 5′-TCTACGAGTGGATGGTGCGTT-3′ and FOXO3-R: 5′- CGACTATGCAGTGACAGGTTGTG-3′.

### Chromatin immunoprecipitation (ChIP)

The cell lines were transfected with pCMV5-FOXK2WT, pCMV5-FOXK2 K527/633 R and the empty vector pCMV5 for 24 h, after which FOXK2-overexpressing cells were collected for the ChIP assay, as previously described^9^. For the immunoprecipitation, 4 µg of either IgG (P0447, Dako; Cambridgeshire, UK) and FOXK2 (ab5298; Abcam; Cambridge, Massachussetts, USA) antibodies were added to the precleared samples. DNA quantification was performed using the picogreen reagent (Life Technologies) and fluorescence was measured in a PHERAstar^Plus^ microplate reader (BMG Labtech, Ortenberg, Germany). For PCR reaction, we used 2.5 µL DNA from each sample, 0.5 µL of mix of primers (50 nM final concentration), 5 µL SYBR green master mix (Applied Biosystems) and 2 µL DEPC-treated water per well. The reaction was run in 7900 HT Fast Real-time PCR System(Applied Biosystems) and the cycling program was 95 °C for 10 min followed by 40 cycles of 95 °C for 15 s, 60 °C for 30 s and 95 °C for 30 s, followed by a dissociation step. The pair of primers used for ChIP was: FOXO3-F, 5′-ACCAACATCTTCGCCGTTC-3′ and FOXO3-R, 5′-GGTGTCCGGTTCCCTGTTAG-3′. All experiments were done in triplicates and results were normalized to the IgG antibody.

### Statistical analysis

Statistical analysis of data was conducted using the SPSS Statistics software (SPSS for Windows, version 17.0, SPSS Inc.). The Student’s *t*-test was used to compare differences between means from two groups. The values of *p < 0.05; **p < 0.01 and ***p < 0.005 were considered statistically significant.

## Electronic supplementary material

Supplementary Figure S1

Supplementary Figure S2

Supplementary Figure Legends

## References

[1] Kaestner KH, Knochel W, Martinez DE (2000). Unified nomenclature for the winged helix/forkhead transcription factors. Genes Dev..

[2] Golson ML, Kaestner KH (2016). Fox transcription factors: from development to disease. Development.

[3] Lam EW, Brosens JJ, Gomes AR, Koo CY (2013). Forkhead box proteins: tuning forks for transcriptional harmony. Nat. Rev. Cancer.

[4] Li C, Lai CF, Sigman DS, Gaynor RB (1991). Cloning of a cellular factor, interleukin binding factor, that binds to NFAT-like motifs in the human immunodeficiency virus long terminal repeat. Proc. Natl. Acad. Sci. USA.

[5] Ji Z (2012). The forkhead transcription factor FOXK2 promotes AP-1-mediated transcriptional regulation. Mol. Cell Biol..

[6] Ji Z (2014). The forkhead transcription factor FOXK2 acts as a chromatin targeting factor for the BAP1-containing histone deubiquitinase complex. Nucleic Acids Res.

[7] Liu Y (2015). FOXK2 transcription factor suppresses ERalpha-positive breast cancer cell growth through down-regulating the stability of ERalpha via mechanism involving BRCA1/BARD1. Sci. Rep..

[8] Shan L (2016). FOXK2 Elicits massive transcription repression and suppresses the hypoxic response and breast cancer carcinogenesis. Cancer Cell.

[9] Nestal de Moraes G (2015). Forkhead box K2 modulates epirubicin and paclitaxel sensitivity through FOXO3a in breast cancer. Oncogenesis.

[10] Chymkowitch P, Nguea PA, Enserink JM (2015). SUMO-regulated transcription: challenging the dogma. Bioessays.

[11] Wilkinson KA, Henley JM (2010). Mechanisms, regulation and consequences of protein SUMOylation. Biochem J..

[12] Myatt SS (2014). SUMOylation inhibits FOXM1 activity and delays mitotic transition. Oncogene.

[13] Danciu TE (2012). Small ubiquitin-like modifier (SUMO) modification mediates function of the inhibitory domains of developmental regulators FOXC1 and FOXC2. J. Biol. Chem..

[14] Sutinen P, Rahkama V, Rytinki M, Palvimo JJ (2014). Nuclear mobility and activity of FOXA1 with androgen receptor are regulated by SUMOylation. Mol. Endocrinol..

[15] Zhao Q (2014). GPS-SUMO: a tool for the prediction of sumoylation sites and SUMO-interaction motifs. Nucleic Acids Res.

[16] Matunis MJ, Coutavas E, Blobel G (1996). A novel ubiquitin-like modification modulates the partitioning of the Ran-GTPase-activating protein RanGAP1 between the cytosol and the nuclear pore complex. J. Cell Biol..

[17] Sunters A (2003). FoxO3a transcriptional regulation of Bim controls apoptosis in paclitaxel-treated breast cancer cell lines. J. Biol. Chem..

[18] Sunters A (2006). Paclitaxel-induced nuclear translocation of FOXO3a in breast cancer cells is mediated by c-Jun NH2-terminal kinase and Akt. Cancer Res.

[19] Karadedou CT (2012). FOXO3a represses VEGF expression through FOXM1-dependent and -independent mechanisms in breast cancer. Oncogene.

[20] Khongkow P (2016). Paclitaxel targets FOXM1 to regulate KIF20A in mitotic catastrophe and breast cancer paclitaxel resistance. Oncogene.

[21] Dou H, Huang C, Van Nguyen T, Lu LS, Yeh ET (2011). SUMOylation and de-SUMOylation in response to DNA damage. FEBS Lett..

[22] Marais A (2010). Cell cycle-dependent regulation of the forkhead transcription factor FOXK2 by CDK.cyclin complexes. J. Biol. Chem..

[23] Hilgarth RS (2004). Regulation and function of SUMO modification. J. Biol. Chem..

[24] Khongkow P (2014). FOXM1 targets NBS1 to regulate DNA damage-induced senescence and epirubicin resistance. Oncogene.

[25] Krol J (2007). The transcription factor FOXO3a is a crucial cellular target of gefitinib (Iressa) in breast cancer cells. Mol. Cancer Ther..

[26] Freddie CT, Ji Z, Marais A, Sharrocks AD (2007). Functional interactions between the Forkhead transcription factor FOXK1 and the MADS-box protein SRF. Nucleic Acids Res.

[27] Kwok JM (2008). Thiostrepton selectively targets breast cancer cells through inhibition of forkhead box M1 expression. Mol. Cancer Ther..

