# Supplementary material for: SUMOylation modulates FOXK2-mediated paclitaxel sensitivity in breast cancer cells
Source: Oncogenesis. 2018 Mar 13;7(3):29. doi: 10.1038/s41389-018-0038-6 (PMC5852961; doi:10.1038/s41389-018-0038-6)
Supplement: Supplementary file 1 — Supplementary Figure S1 [file 41389_2018_38_MOESM1_ESM.pptx]

## Slide 1
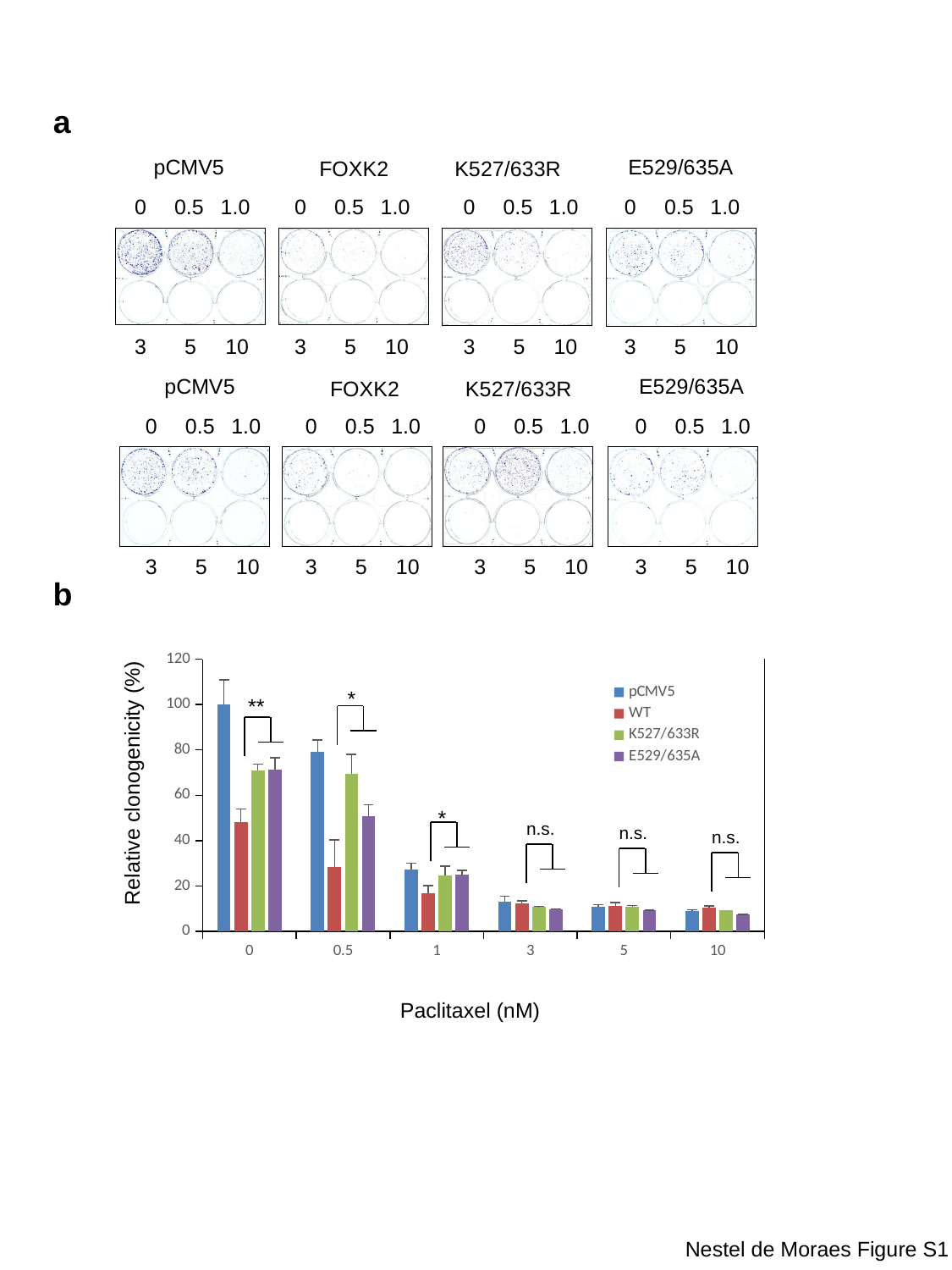

a
pCMV5
E529/635A
FOXK2
K527/633R
0
0.5
1.0
0
0.5
1.0
0
0.5
1.0
0
0.5
1.0
3
5
10
3
5
10
3
5
10
3
5
10
pCMV5
E529/635A
FOXK2
K527/633R
0
0.5
1.0
0
0.5
1.0
0
0.5
1.0
0
0.5
1.0
3
5
10
3
5
10
3
5
10
3
5
10
b
### Chart
| Category | pCMV5 | WT | K527/633R | E529/635A |
|---|---|---|---|---|
| 0.0 | 99.999969169117 | 48.15783932238649 | 70.7568765972324 | 71.37349425821051 |
| 0.5 | 79.1074243541161 | 28.29657829610659 | 69.44656406765407 | 50.66131319213405 |
| 1.0 | 27.36950227547328 | 16.85169543650519 | 24.4951365823534 | 25.06289006847847 |
| 3.0 | 13.07229441273488 | 12.14736792126779 | 10.72914730101825 | 9.603820069733292 |
| 5.0 | 10.83705539168941 | 11.33034952047186 | 10.79080906711606 | 9.264680356195356 |
| 10.0 | 8.971786967230779 | 10.28209949680916 | 9.218434031622005 | 7.414827373261177 |*
**
Relative clonogenicity (%)
*
n.s.
n.s.
n.s.
Paclitaxel (nM)
Nestel de Moraes Figure S1
