# Supplementary material for: SUMOylation modulates FOXK2-mediated paclitaxel sensitivity in breast cancer cells
Source: Oncogenesis. 2018 Mar 13;7(3):29. doi: 10.1038/s41389-018-0038-6 (PMC5852961; doi:10.1038/s41389-018-0038-6)
Supplement: Supplementary file 2 — Supplementary Figure S2 [file 41389_2018_38_MOESM2_ESM.pptx]

## Slide 1
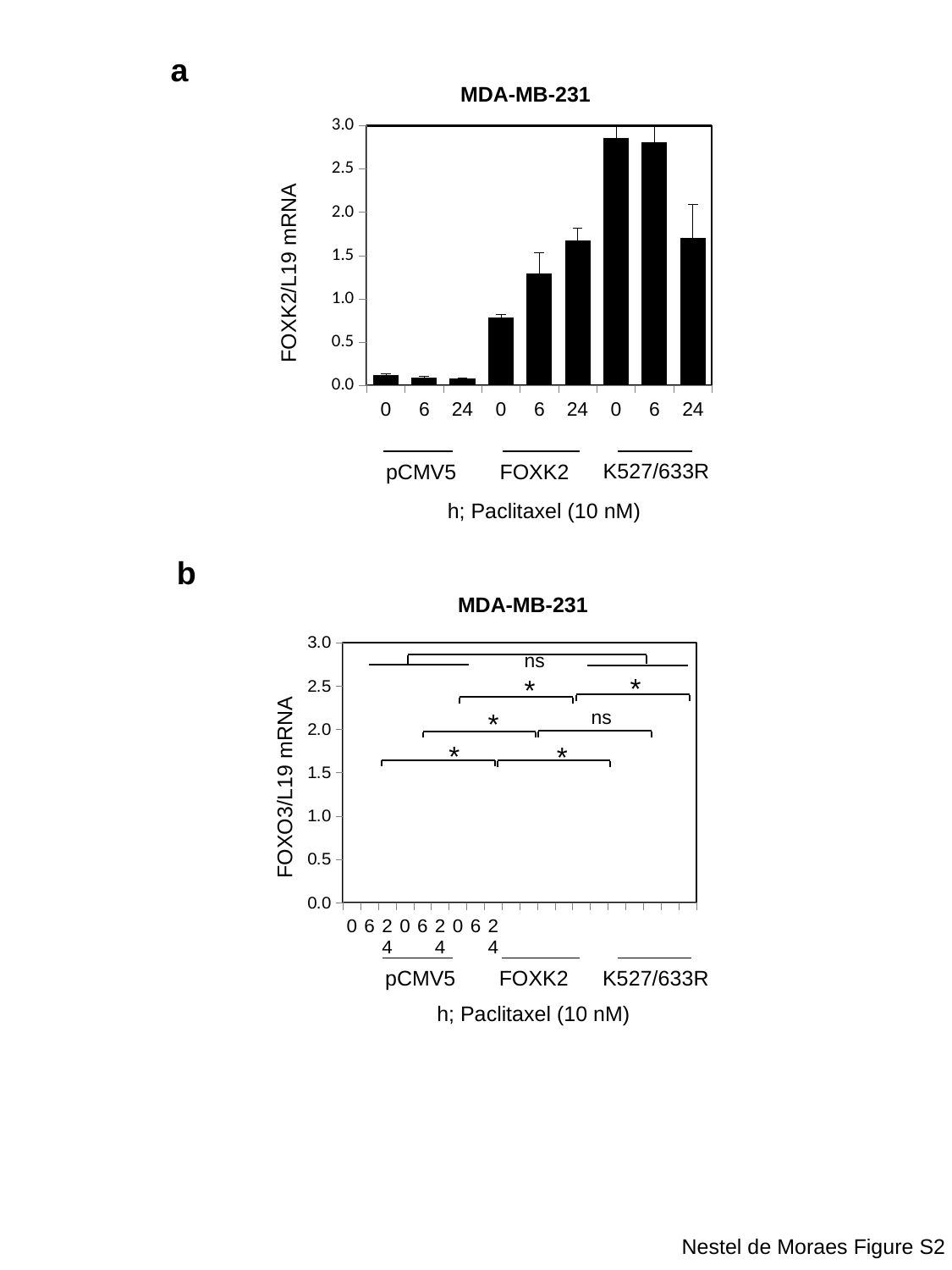

a
MDA-MB-231
### Chart
| Category | |
|---|---|
| 0 | 0.123968564067168 |
| 6 | 0.0953863253897745 |
| 24 | 0.0787517188987127 |
| 0 | 0.782900628387672 |
| 6 | 1.29668067452003 |
| 24 | 1.675754418057363 |
| 0 | 2.856285033617879 |
| 6 | 2.806606278065523 |
| 24 | 1.70224334964444 |FOXK2/L19 mRNA
K527/633R
pCMV5
FOXK2
h; Paclitaxel (10 nM)
b
MDA-MB-231
### Chart
| Category | |
|---|---|
| 0 | 0.834337717071254 |
| 6 | 0.899121687739725 |
| 24 | 0.961871019940484 |
| 0 | 1.173867106342228 |
| 6 | 1.313887516678042 |
| 24 | 1.401917787593976 |
| 0 | 0.959260111076872 |
| 6 | 1.091578251553972 |
| 24 | 0.932571673525719 |ns
*
*
ns
*
*
*
FOXO3/L19 mRNA
K527/633R
pCMV5
FOXK2
h; Paclitaxel (10 nM)
Nestel de Moraes Figure S2
