# Supplementary material for: SUMOylation modulates FOXK2-mediated paclitaxel sensitivity in breast cancer cells
Source: Oncogenesis. 2018 Mar 13;7(3):29. doi: 10.1038/s41389-018-0038-6 (PMC5852961; doi:10.1038/s41389-018-0038-6)
Supplement: Supplementary file 3 — Supplementary Figure Legends [file 41389_2018_38_MOESM3_ESM.docx]

**Supplementary Figure S1**. **Clonogenic assays to study the effects of overexpessing FOXK2 wild-type and SUMO-defective mutants on the cytotoxicity of paclitaxel in MCF-7 cells**. MCF- 7 cells were transfected with the empty vector (pCMV5), wild-type FOXK2 and SUMO-mutant (K527/633R) and (E529/635A) FOXK2 vector, seeded in 6-well plates and treated with increasing concentrations of paclitaxel. After 48 h of incubation with the drugs, cells were culture in fresh media, grown for around 14 days and stained with crystal violet. (a) The images are representative results of > 3 independent experiments. (b) The graphs are representative of three experiments. Statistical significant differences between cells transfected with the wild-type and K527/633R or E529/635A mutant were determined by Student’s t-test (* p≤0.05, **p≤0.01, ***p≤0.001, significant).

**Supplementary Figure S2. Overexpression of the Wild-type but not a SUMO-mutant form of FOXK2 induces FOXO3 mRNA expression.** MDA-MB-231 cells were transfected with the empty vector (pCMV5) wild-type FOXK2 and SUMO-mutant FOXK2 vector (K527/633R) and collected for analysis of FOXK2 (a) and FOXO3 (b) expression by qRT-PCR**.** Bars represent average ± s.d. of three independent experiments. Statistical significance was determined by Student’s t-test (* *p*≤0.05, ***p*≤0.01, ****p*≤0.001, significant).
